# SurgiCal Obesity Treatment Study (SCOTS): protocol for a national prospective cohort study of patients undergoing bariatric surgery in Scotland

**DOI:** 10.1136/bmjopen-2015-008106

**Published:** 2015-05-22

**Authors:** Jennifer Logue, Sally Stewart, Jane Munro, Julie Bruce, Eleanor Grieve, Mike Lean, Robert S Lindsay, Duff Bruce, Abdulmajid Ali, Andrew Briggs, Naveed Sattar, Ian Ford

**Affiliations:** 1 Institute of Cardiovascular and Medical Sciences, University of Glasgow, Glasgow, UK; 2 Institute of Health and Wellbeing, University of Glasgow, Glasgow, UK; 3 Clinical Trials Unit, University of Warwick, Warwick, UK; 4 School of Medicine, University of Glasgow, Glasgow, UK; 5 NHS Grampian, Aberdeen, UK; 6 University Hospital Ayr, NHS Ayrshire and Arran, Ayr, UK; 7 Robertson Centre for Biostatistics, University of Glasgow, Glasgow, UK

**Keywords:** NUTRITION & DIETETICS, EPIDEMIOLOGY, HEALTH ECONOMICS

## Abstract

**Introduction:**

The efficacy of bariatric surgery for large-scale, long-term weight loss is well established. However, many questions remain over the continual benefits and cost-effectiveness of that weight loss for overall health, particularly when accounting for potential complications and adverse events of surgery. Health research institutes in the UK and the USA have called for high-quality longitudinal cohort studies of patients undergoing bariatric surgery, assessing outcomes such as surgical complications, mortality, diabetes remission, microvascular complications, cardiovascular events, mental health, cost and healthcare use.

**Methods and analysis:**

SurgiCal Obesity Treatment Study (SCOTS) is a national, prospective, observational, cohort study of patients undergoing primary bariatric surgical procedures in Scotland. This study aims to recruit 2000 patients and conduct a follow-up for 10 years postbariatric surgery using multiple data collection methods: surgeon-recorded data, electronic health record linkage, and patient-reported outcome measures. Outcomes measured will include: mortality, weight change, diabetes, surgical, cardiovascular, cancer, behavioural, reproductive/urological and nutritional variables. Healthcare utilisation and economic productivity will be collected to inform cost-effectiveness analysis.

**Ethics and dissemination:**

The study has received a favourable ethical opinion from the West of Scotland Research Ethics committee. All publications arising from this cohort study will be published in open-access peer-reviewed journals. All SCOTS investigators (all members of the research team at every recruiting site) will have the ability to propose research suggestions and potential publications using SCOTS data; a publications committee will approve all requests for use of SCOTS data and propose writing committees and timelines. Lay-person summaries of all research findings will be published simultaneously on the SCOTS website (http://www.scotsurgeystudy.org.uk).

**Strengths and limitations of this study**

- The major advantage of SurgiCal Obesity Treatment Study (SCOTS) is the availability of high-quality health record data recorded in a standardised manner across Scotland.
- Inclusion of all state-funded and private hospitals undertaking bariatric surgery ensures geographical representation.
- SCOTS will allow detailed follow-up of clinical, surgical outcomes and changes in health status after bariatric surgery, underpinned with a detailed cost-effectiveness analysis based on real-life care and treatment practices.
- The design of the study means that participants will be able to be followed through record-linkage for many decades, until death, at minimal additional cost. All patients can be followed, therefore removing any potential for bias from/to participant drop-out due to poor surgical outcome or ill health.
- The major limitation of SCOTS is the relatively low numbers of bariatric surgery procedures performed in Scotland per head of population, and the challenge of low volume centres compared with international standards. However, the data collection has been designed with this in mind to ensure that surgeon and centre experience is captured, and this variety will allow surgical experience to be explored as an explanatory variable.

## Introduction

The efficacy of bariatric surgery for large-scale, long-term weight loss is well established.^1^ However, many questions remain over the continual benefits of that weight loss for overall health, particularly when accounting for potential complications of surgery. In 2009, Picot *et al*^2^ published a systematic review and economic evaluation of the clinical and cost-effectiveness of bariatric surgery for obesity, funded by the UK's National Institute for Health Research (NIHR) Health Technology Assessment Programme (HTA). The conclusion was that high-quality data were lacking and further research was required, particularly large-scale cohort studies to ascertain patient quality of life, late complications requiring reoperation, the effect of surgeon experience, duration of comorbidity remission and the full use of health and care resources to allow comprehensive cost-effectiveness analysis.

In May 2013, the USA National Institute for Diabetes and Digestive and Kidney Diseases and the National Heart, Lung and Blood Institute held a multidisciplinary workshop on the long-term outcomes of bariatric surgery, and reached similar conclusions regarding research priorities to that of Picot *et al.*^3^ The main areas highlighted as having significant knowledge gaps included the incidence of surgical complications, overall mortality, duration of type 2 diabetes remission and microvascular complications, cardiovascular events, mental health outcomes, malignancies, fertility and fetal outcomes, and overall cost and healthcare use. This workshop agreed that there should be investment in high-quality, observational studies, potentially utilising electronic health records where available.

In response to the Picot systematic review, in 2010, UK NIHR HTA issued an open call for research proposals for a long-term longitudinal cohort study of patients undergoing bariatric surgery in the UK, with a minimum of 10 years follow-up. The outcomes were to include quality of life, survival, weight, BMI, diabetes and surgical complications. The SurgiCal Obesity Treatment Study (SCOTS) was funded in 2011. This paper describes the study design and outcomes.

## Objectives

The overall objective of SCOTS is to establish the clinical outcomes and adverse events of different bariatric surgical procedures, their impact on quality of life and nutritional status, and the effect on comorbidities in the short and long term in a cohort of over 2000 patients.

The specific objectives are to establish in a cohort of obese patients who are undergoing bariatric surgery

- All-cause and cause-specific mortality over a mean of 10 years since bariatric surgery.
- Incidence of cardiovascular disease, cancer and diagnosis of diabetes over a mean of 10 years since bariatric surgery.
- Incidence of acute and chronic postoperative complications. Acute complications, defined as up to 3 months postsurgery, will include surgical site infection; chronic complications will include revisional surgery, plastic surgery and chronic pain.
- Change in health-related quality of life, anxiety and depression over time, preoperatively and postoperatively, for a mean of 10 years from the date of bariatric surgery.
- The micronutrient and weight status preoperatively and postoperatively for a mean of 10 years since bariatric surgery.
- The glycaemic control, lipids, blood pressure, medication prescription and rate of diabetes complications (microalbuminuria and renal disease, retinopathy and foot ulceration) in those that have pre-existing diabetes or develop diabetes during a mean of 10 years of follow-up since bariatric surgery.
- Explanatory variables will be explored for each outcome.

## Methods

### Study design

SCOTS is a national, prospective, observational cohort study of patients undergoing primary bariatric surgical procedures in Scotland. A total of 2000 patients will be recruited over a 5-year period from 2014 to 2019, and will be followed for a mean follow-up period of 10 years postsurgery.

### Participant and centre eligibility

Patients scheduled to undergo bariatric surgery at any state-funded or private hospital in Scotland will be eligible for entry into the cohort. Currently, 10 state-funded and 4 private hospitals across Scotland provide bariatric surgery, and are thus eligible to participate.

For the purposes of SCOTS, a bariatric surgical procedure is defined as ‘any surgical intervention which has the primary purpose of large-scale weight loss in a patient who is obese’. Eligible procedures include Roux-en-Y gastric bypass (RYGB), sleeve gastrectomy (SG) and laparoscopic adjustable gastric banding (LAGB). The inclusion of emerging techniques in this definition will be considered by the Steering Committee throughout the duration of the cohort. At current rates for Scotland, it is anticipated that this may result in 60% of participants having SG, 30% LAGB, and 20% RYGB, but changing trends in the type of surgery offered may alter these predicted patterns.

SCOTS has received a favourable ethical opinion from the West of Scotland Research Ethics Committee, and full management approval at each hospital site.

### Outcomes

The outcomes to be measured include mortality, weight, diabetes, surgical complications, cardiovascular, cancer, behavioural, reproductive/urological and nutritional outcomes. Other healthcare resource use of variables will be collected to inform a cost-effectiveness analysis. Outcomes are summarised in table 1.

**Table 1:** Surgical obesity treatment study outcomes

Outcome category	Specific outcomes
Mortality	30 day, 1 year and 10 year mortality; all-cause and cause-specific
Weight	Change in weight/body mass index
Surgical	Incidence of surgical complications 1 year and 10 years postoperatively
	Hospital length of stay/rate of readmission
	Incidence of surgical site infection
	Postoperative pain
	Rate of reoperation
	Rate of revisional procedures
	Rate of body-contouring surgery
	Incidence of excess skin affecting daily life
	Change in gastrointestinal symptoms
Cardiovascular	Incidence of fatal and non-fatal cardiovascular events
	Incidence of fatal and non-fatal coronary heart disease
	Cardiovascular medication prescription
Diabetes	Incidence of type 2 diabetes
	Change in glycaemic control
	Change in blood lipid concentration
	Change in blood pressure
	Change in estimated glomerular filtration rate and microalbuminuria
	Incidence of retinopathy
	Incidence of amputation
	Incidence of chronic kidney disease
	Diabetes medication prescription
Cancer	Incidence of all and specific cancers
Behavioural	Change in prevalence/incidence of depression and anxiety
	Change in health-related quality of life
	Change in rate of alcohol use disorder
	Change in smoking status
	Change in physical activity levels
	Change in fat, fibre and calcium intake
	Life orientation (optimism)
Reproductive/urological	Change in menstrual cycle/abnormalities
	Incidence of pregnancy and fetal outcome
	Change in prevalence of incontinence
	Change in prevalence of prostatic symptoms
	Change in rate of erectile dysfunction
Nutritional	Incidence of micronutrient deficiency
	Prescription of multivitamins and nutritional supplements
Economic	Cost of presurgical and postsurgical care pathways
	Healthcare utilisation
	Change in receipt of Social Security benefits
	Change in employment status
	Change in education
	Sickness absence from work/study
	Change of use of aids and specialist equipment (eg, walking frame or stair lift)
	Social care requirements
Fractures	Incidence of fractures by site

### Data collection

Data will be obtained from three main sources: the surgical team, patients, and from electronic health records (table 2). For some outcomes, multiple sources will be used to collect similar data to ensure overall completeness of the data set (eg, revisional surgery will be collated from clinical teams, self-reported by patients and from electronic health records; fractures will be reported through either hospital and emergency department admissions, or through radiology reports).

**Table 2:** Surgical obesity treatment study data collection

Data collection	Type	Questionnaire
Surgical data		
	Primary procedure	Surgeon
		Procedure
		Operative details (including band type, staple type, approach, dissection, biliopancreatic limb length)
		Presurgical intervention (including balloon or endobarrier)
		Patient weight day of operation
		American Society of Anesthesiologists (ASA) grade
		Operation performed by surgeon in training
		Perioperative blood transfusion
	Reoperations	Reoperation (detail as per primary procedure)
		Reason for reoperation
	Surgical experience	Surgeon experience (baseline, updated annually)
Patient-reported outcome measures		
	Demographics	ID, sex, age
		Marital status
		Ethnicity
		Education
		Occupation
	Medical history	Medical history^8^
		Weight history^9^
		Family history (diabetes, coronary heart disease (CHD))
		Family history (weight)
	Quality of life—Health related	SF12^10^
		EQ5D 5 L^11^
		Patient Health Questionnaire (PHQ9)^12^
		Generalised Anxiety Disorder (GAD7)^13^
	Impact of weight	Impact of Weight on Quality of Life (IWQOL)-Lite^14^
		Reproductive health (female)^5^
		Erectile dysfunction—Massachusetts Male Aging Study: Single question assessment (male)^15^
		International Prostate Symptom Score^16^
		International Consultation on Incontinence Questionnaire Urinary Incontinence Short Form^17^
		Modified Reflux questionnaire^18^
	Lifestyle	Smoking
		Alcohol Use Disorders Identification Test: Self-Report Version^19^
		International Physical Activity Questionnaire short last 7 days self-administered format^20^
		Dietary Fat and Fibre intake
		Dietary Intervention in Primary Care (DINE)^21^
	Personal outlook	Life orientation test (revised)^22^
		Expectations of surgery—Goals and Relative Weights Questionnaire^23^
	Surgical recovery	Postoperative pain
		Surgical site infection^24^
		Skin excess
		Body contouring surgery^25^
	Healthcare usage	Outpatient care^26^
		Contacts with other health and social care professionals
		Devices and specialist equipment^27^
		Benefits
		Multivitamins and supplements use
Electronic health records	Hospital admissions	Diagnosis, length of stay, intensive/high dependency, in-hospital mortality
		Scottish Morbidity Record 01
	Maternity	Inpatient and day case including birth weight, gestational age, mode of delivery, induction, pregnancy outcome and neonatal care
		Scottish Morbidity Record 02/Scottish Birth Record
	Psychiatric	Inpatient care records—including diagnosis, treatment, length of stay
		Scottish Morbidity Record 04
	Malignancy	Site, histology, stage, grade, treatment
		Scottish Cancer Registry
	Mortality	National Records of Scotland Death Records
	Diagnostic tests	Biochemistry, haematology, radiology, pathology and microbiology results
		Scottish Care Information—Store
	Diabetes	Including date of diagnosis, type, treatment, retinal and foot screening
		Scottish Care Information—diabetes
	Medications	Type and adherence—encashed prescriptions
		Prescribing Information System

Where possible, definitions and data sources have been kept consistent with the UK National Bariatric Surgery Register^4^ and the NIH-funded Longitudinal Assessment of Bariatric Surgery^5^ to allow comparison and meta-analysis of outcomes.

#### Recruitment procedures and consent

Patients are approached about the study at least 4 weeks prior to their primary bariatric surgical procedure. This is undertaken by either the clinical bariatric surgery team or by a research nurse in preoperative assessment clinics. Patients are shown an 8 min information film and provided with an information booklet explaining the SCOTS. Individual participant consent is obtained on a subsequent clinical visit. Informed consent is sought at three levels, for (1) access to medical records and linkage of their electronic health records, (2) postal questionnaires preoperatively and postoperatively and (3) contact for future studies. Each level of consent is dependent on the previous level, for example, postal (or electronic) questionnaires are not sent without consent to link electronic health records.

#### Surgical data

In order to encourage site participation and maintain interest in the study, data from the surgical clinical team is collected using a specifically designed, web-based, clinical information system which has a number of features of use to the clinical teams. At first log-in, the system records basic surgical data, including surgeon experience, local operative procedures performed at that site including the standard approach for each procedure performed by that surgeon (eg, make of staple or bands used). Details of consented individuals are entered by the central study centre, and the details of each patient are visible to that local clinical team allowing them to enter further preoperative and surgical details. This is kept simple by allowing some modification to the standard surgical details stored. SCOTS data collection system allows weight and band adjustments at follow-up visits to be recorded, as well as details of, and indications for, revisional surgery. If revisional surgery is being performed at another site, the patient's record can be identified on the system. A number of templates exist for clinical letters, patient reports and clinical notes which populate with details from the system and output as a portable document format (pdf) file for entry into hospital letters and records.

#### Patient-reported outcome measures

Participants are asked to complete a suite of questionnaires at a number of time points throughout the study: preoperatively and 1 month, 6 months, 1, 2, 3, 5, 7 and 10 years postoperatively. Questionnaires were developed in conjunction with patient involvement and were piloted (described below). Final questionnaires consist of validated scales covering a wide range of outcome measures. Participants have the option of completing these via a web-based system or on paper by post. They receive up to two reminders for each questionnaire. The full questionnaire takes around 1 h to complete, and start and finish dates are recorded to allow them to be completed over several days.

Questionnaires at all time points include weight, marital status, education, employment, general and obesity-specific quality of life, anxiety, depression, reproductive health, urological health, gastrointestinal symptoms, smoking, alcohol use, physical activity, diet, healthcare utilisation, social security and nutritional supplements. Baseline preoperative questionnaires include demographics, medical history, family history, weight history and expectations of surgery. Postoperative questionnaires include surgical recovery, pain intensity, surgical site infection, skin excess and plastic surgery.

#### Electronic health records

Scotland is fortunate to have excellent electronic health records. All patient healthcare interactions in Scotland are recorded by use of a single patient ID number. IT systems are common across all 14 health board areas, and a single government-funded department (Information Services Division) collates information for research purposes. This has allowed Scotland to become a leader in research using routine health records, and these systems are being utilised for follow-up in SCOTS.

Participants in SCOTS will be followed using multiple existing Scottish health record data sets: Scottish Morbidity Records—general acute/inpatient and day case (SMR 01), maternity inpatient and day case (SMR 02), and mental health inpatient and day case (SMR 04) data sets; National Records of Scotland Death Records; Scottish Cancer Registry; Scottish Care Information diabetes data set (SCI-Diabetes); Scottish Care Information Store (SCI-Store) diagnostic data set and the prescribing information system dataset.

Evidence of quality assurance for electronic health record data sets in Scotland is very high: an assessment of 2010/2011 data by comparison with local hospital case records showed an overall high degree of accuracy. For conditions of interest, this included accuracy of 97.3% for ischaemic heart disease, 99.3% for fractures, 98.2% for myocardial infarction and 98.4% for upper gastrointestinal endoscopy.^6^ Diabetes records are derived from all hospitals in Scotland and all of approximately 1000 primary care centres (except 5), which are downloaded to SCI-Diabetes on a daily basis. This dataset represents over 99% of people with diagnosed diabetes in Scotland.

#### Treatment pathways

Every site completes an annual survey to detail their current preoperative and postoperative care pathways. This includes how patients enter the surgical pathway (eg, direct or self-referral or via a non-surgical weight management programme), any presurgical requirements for weight loss and the standard numbers of appointments with dieticians, psychologists, surgeons and physicians that each patient receives. This information will allow outcomes from high-contact and low-contact presurgical and postsurgical care pathways to be compared.

#### Health economics

Health economist involvement in the design and development of the study has ensured that all relevant healthcare resource use data will be captured. Cost analysis will involve comparison of preoperative and postoperative care pathways at each site, and will include estimates of the costs of immediate (30 day) postoperative complications.

#### Surgical learning curve

The effect of the surgical learning curve on patient outcome is now well recognised.^7^ In order to account for this within analyses, surgeons will be asked to record date of completion of postgraduate surgical training and volume of bariatric surgery procedures performed (total, last 1 and 5 years). Total numbers of bariatric procedures (primary and revisional) performed by each individual surgeon in the previous year are also requested annually.

#### Adverse outcome reporting

Given the duration of this epidemiological study, there is a possibility that higher than expected adverse event rates may be detected, such as surgical site infection or 30-day surgical mortality. An adverse outcome plan has been agreed by the Steering Committee, and all participating surgeons have approved this plan. In preparation for each peer-reviewed publication, the study statisticians will generate reports to assess variability in outcomes between study sites, but these will be anonymised. No statistical analysis will be undertaken at individual surgeon level. Rates will be presented for all sites using funnel plots, with adjustment for case-mix, to identify sites that have outcome rates significantly above or below what would be expected given patient characteristics (which will be predefined in the statistical analysis plan). If a particular site is identified by the SCOTS team as having a potentially poorer outcome than expected, a letter will be sent from the Chief Investigator to the Principal Investigator and the Medical Director covering that site. An individual report will be prepared for that study site, summarising key data for that site, and including copies of the funnel plots from the main report, with the data point for that study site highlighted. Outcomes which will be considered eligible for assessment include rates of mortality, pulmonary thromboembolic disease, cardiovascular disease at 1 year, reoperations, surgical site infections, and nutritional deficiencies.

### Reference populations/comparator groups

Collection of similar data from a non-surgical comparator group is not funded within the SCOTS, however, we have access to a number of possible reference populations. Electronic diabetes health record data sets allow an anonymous comparator group, matched for key demographics, to be created with prospective follow-up of diabetes care and complications compared to those with diabetes who had bariatric surgery. Also, several of the non-surgical weight management services within Scotland have detailed patient records again allowing the formation of anonymous comparator groups. Large cohorts, such as UK Biobank, also provide reference populations with prospective outcomes. In time, we hope to develop a similar cohort of patients undergoing lifestyle weight management with a comparable collection of baseline data, including psychological and mental health questionnaires, to help account for any biases within a self-selecting bariatric surgery population.

### Patient involvement

We sought to form a patient public involvement (PPI) group to allow patient representation and input in the design of the study; public and patient involvement is encouraged by UK research councils, and mandatory for NIHR-funded research. A small focus group was formed comprising five patients who had previously undergone bariatric surgery in either the state-funded or private sector. The SCOTS PPI group has been consulted over a number of topics including: the information provided to patients about the study (patient information sheets, invitation letters and web site), content and design of electronic and paper patient-reported questionnaires, communication throughout the study, such as newsletters and reminder letters, and the need for incentives to take part and complete questionnaires. The focus group has met twice, each meeting lasting 3–4 h; participants have also been contacted by email periodically with additional questions.

### Development of patient questionnaires

Piloting of patient questionnaires was undertaken during the development phase of the study. Some specific questions were considered by researchers to be of a sensitive nature (eg, sexual function, continence). However, these were not considered ‘sensitive’ by the SCOTS PPI focus group. Rather, items relating to diet, exercise and welfare benefits were considered to be particularly sensitive because they felt participants may feel ‘judged’. To counter this, the PPI group requested the inclusion of a short explanatory sentence on why sections of questions were included. The group also identified important topics that were not included within the original question set, in particular, the inclusion of the need for dental work after bariatric surgery. Incentives were not felt to be necessary and were discounted in favour of a six monthly newsletter to include study progress, findings and words of continued thanks for participation.

## Dissemination plans

All publications arising from this cohort will be published in open-access peer-reviewed journals. All SCOTS investigators (all members of the research team at every recruiting site) will have the ability to propose research suggestions and potential publications using SCOTS data; a publications committee will approve all requests for use of SCOTS data and propose writing committees and timelines. Lay-person summaries of all research findings will be published simultaneously on the SCOTS website (http://www.scotsurgeystudy.org.uk). As is now recognised practice, we plan to make our data fully available for use in an anonymous form by other researchers at the end of the currently funded time period of this study (2027). Patient consent procedures cover the sharing of data for future research by external researchers. We will follow the NHS code of Practice on Protecting Patient Confidentiality issued by NHS Scotland. We will require a written request/proposal for data to be made to our publications committee who will need to be satisfied that all ethical approvals are in place and that funds are available to cover any costs of collating the necessary data. Regular reports of progress with the research will be required. The collaboration with the SCOTS study group should be acknowledged in the manuscript, normally by authorship stating the research workers ‘on behalf of the SCOTS group’.

## Advantages and limitations

The major advantage of SCOTS is the availability of high-quality health record data recorded in a standardised manner across Scotland. Inclusion of all state-funded and private hospitals undertaking bariatric surgery ensures geographical representation.

SCOTS will allow detailed follow-up of clinical, surgical outcomes and changes in health status after bariatric surgery, underpinned with a detailed cost-effectiveness analysis based on real-life care and treatment practices. The design of the study means that participants will be able to be followed through record-linkage for many decades, until death, at minimal additional cost. All patients can be followed, therefore removing any potential for bias from to participant dropout due to poor surgical outcome or ill health.

The major limitation of SCOTS is the relatively low numbers of bariatric surgery procedures performed in Scotland per head of population, and the challenge of low-volume centres compared with international standards. As a consequence, RYGB is the least commonly performed operation. These factors will be taken into account during analysis and compared with results from other studies. However, the data collection has been designed with this in mind to ensure that surgeon and centre experience is captured, and this variety will allow surgical experience to be explored as an explanatory variable.

## Conclusions

SCOTS is the first national epidemiological study investigating long-term outcomes after bariatric surgery. This large data set will be one of the most detailed prospective cohort studies of bariatric surgery to date, made possible through linkage to high-quality, country-wide, electronic health records. SCOTS will assess important clinical, surgical and patient-reported outcomes over a 10-year period, including the incidence of, and risk factors for, surgical complications, overall mortality, diabetes remission, diabetes, microvascular complications, cardiovascular events, mental health, cancers, genitourinary and reproductive outcomes. This study will add substantially to the evidence base for the long-term effectiveness and cost-effectiveness of bariatric surgery outcomes.

### International implications

No single study should be used to generate evidence for a commonly performed group of procedures that are the most effective treatment for a condition that effects ∼28% of people in the USA, and ∼22% of Europeans. We have developed a data set to allow important questions about bariatric surgery to be answered. However, bariatric surgery in Scotland is performed in low numbers compared with other countries; while we have excellent electronic data linkage, overall numbers of patients are low. Other sites may not have access to similar linked data, but do have higher numbers of patients. We would welcome the opportunity to collaborate with sites in other countries to compare patient-reported data, and electronic health record data when available, to validate the generalisability of our detailed clinical outcome data for other populations.
